# Single-administration, thermostable human papillomavirus vaccines prepared with atomic layer deposition technology

**DOI:** 10.1038/s41541-020-0195-4

**Published:** 2020-06-02

**Authors:** Robert L. Garcea, Natalie M. Meinerz, Miao Dong, Hans Funke, Saba Ghazvini, Theodore W. Randolph

**Affiliations:** 1 The BioFrontiers Program, University of Colorado, Boulder, CO USA; 2 Department of Molecular, Cellular, Developmental Biology, University of Colorado, Boulder, CO USA; 3 Department of Chemical and Biological Engineering, University of Colorado, Boulder, CO USA

**Keywords:** Biotechnology, Immunology

## Abstract

Cold-chain requirements affect worldwide distribution of many vaccines. In addition, vaccines requiring multiple doses impose logistical and financial burdens, as well as patient compliance barriers. To address such limitations, we have developed new technologies to prepare thermostable, single-shot, prime-boost microparticle vaccines. Antigen/adjuvant formulations containing glass-forming polymers and trehalose first are spray-dried to form glassy microparticles that confer thermostability. Atomic layer deposition (ALD) reactions conducted in fluidized beds are then used to coat the microparticles with defined numbers of molecular layers of alumina that modulate the timed release of the internalized antigen and act as adjuvants. We have used a model HPV16 L1 capsomere antigen to evaluate the properties of these technologies. Thermostabilized powders containing HPV16 L1 capsomeres were prepared by spray-drying, coated by ALD with up to 500 molecular layers of alumina, and injected into mice. Antigen distribution was assessed by live-animal IR dye tracking of injected labeled antigen. Antibody responses were measured weekly by ELISA, and neutralizing antibodies were measured by pseudovirus neutralization assays at selected time points. Thermostability was evaluated by measuring antibody responses after incubating ALD-coated antigen powders for one month at 50 °C. Single doses of the ALD-coated vaccine formulations elicited a prime-boost immune response, and produced neutralizing responses and antibody titers that were equivalent or superior to conventional prime-boost doses of liquid formulations. Antibody titers were unaffected by month-long incubation of the formulations at 50 °C. Single-dose, thermostable antigen preparations may overcome current limitations in HPV vaccine delivery as well as being widely applicable to other antigens.

## Introduction

The practical impacts of vaccines are often compromised by common challenges faced in their delivery to patients. In particular, instability of currently licensed vaccines during their storage and handling requires sustained refrigeration^1–4^, and the need for multiple doses can reduce patient compliance. We have developed two technologies that when combined address vaccine storage and cold-chain requirements by increasing thermostability and also encourage patient compliance by requiring only single-shot administration. In order to achieve these objectives, we combined two processes: spray-drying to produce highly thermostable powder preparations of the antigen in glassy matrices composed of disaccharides and polymers, and atomic layer deposition (ALD) processing to coat the stabilized antigen in these core particles with precise nanoscopic layers of alumina whose dissolution can be tuned to deliver booster doses at defined times after administration.

The first technology derives from recently developed methods to lyophilize antigens with adjuvants to create vaccines with superior thermostability while maintaining robust immunogenicity. Immunogens and adjuvants are embedded in glassy organic matrices formed from disaccharide-containing mixtures by adjusting lyophilization and formulation parameters in order to control nucleation rates, glass transition temperatures, and other material properties^5^. We and others have recently demonstrated in murine and non-human primate models that this technology can be successfully applied to provide thermostable formulations of vaccines against ricin toxin^6,7^, anthrax^8,9^, botulinum toxin^10^, ebola glycoprotein^11^ and human papillomavirus^12^. Briefly, the process uses controlled, rapid freezing rates combined with addition of relatively high concentrations of formulation excipients such as trehalose or sucrose that rapidly form glasses upon freezing. When these glasses are dried during a lyophilization process, the resulting dry glass powders, which contain embedded antigens, adjuvants, and coadjuvants, become rigid and exhibit slow internal molecular motions. In turn, protein physical and chemical degradation pathways that require molecular motion are inhibited, as are other vaccine degradation pathways such as the agglomeration of adjuvant nanoparticles. We have now optimized these formulations to include starch polymers that raise glass transition temperatures and extend the process to allow spray-drying of these formulations to form glass-phase, spherical microparticles in which antigens and adjuvants are encased.

The second technology uses ALD techniques^13–15^. ALD allows deposition of nanometer-thick layers of alumina on the spherical surface of the thermally stabilized, antigen-containing microparticulate powders produced by spray-drying. The ALD process applies multiple cycles of sequential, self-limiting reactions in fluidized bed reactors. Each cycle of sequential reactions deposits a single, 2.3-Å-thick, conformal layer of alumina (Al_2_O_3_) on the spherical microparticle surfaces. The number of cycles can be specified, providing control over the layer thickness to within a few Å. With multiple cycles, alumina layers that are 100–500 nm (or greater) thick can be applied to the surfaces. These nanoscopic alumina layers serve multiple functions. When the alumina-coated antigen particles are injected in vivo, the coating dissolves slowly, providing a time-delayed booster dose of antigen. The deposited alumina coatings serve as an adjuvant, replacing the common alum adjuvants. The amorphous alumina coatings produced by ALD are impervious to water vapor, and thus may protect the antigens within the microparticle core from damage resulting from inadvertent water exposure (e.g., water vapor that can be transported from vial stoppers^16^) that can destabilize conventional lyophilized powders during long-term storage^17^.

In the current study we have used the human papillomavirus type 16 (HPV16) L1 capsid protein as a model antigen for evaluating these technologies. This protein antigen has been previously characterized immunologically, and when conformationally intact it induces neutralizing antibodies in murine models^18,19^. This antigen was studied previously using lyophilization to demonstrate retention of conformational integrity and thermostability after high temperature storage of the lyophilized powders^12^. We have now extended these findings to thermostable spray-dried powders of L1 capsomeres that have undergone ALD of alumina on their surface. We show that these ALD-coated antigen preparations elicit a prime-boost immune response to the L1 antigen after a single administration, with antibody titers meeting or exceeding those seen with a standard, alum-adsorbed two-dose immunization of the L1 protein.

## Results

### Physical characteristics of particles after spray-drying and atomic layer deposition

**Fig. 1:**
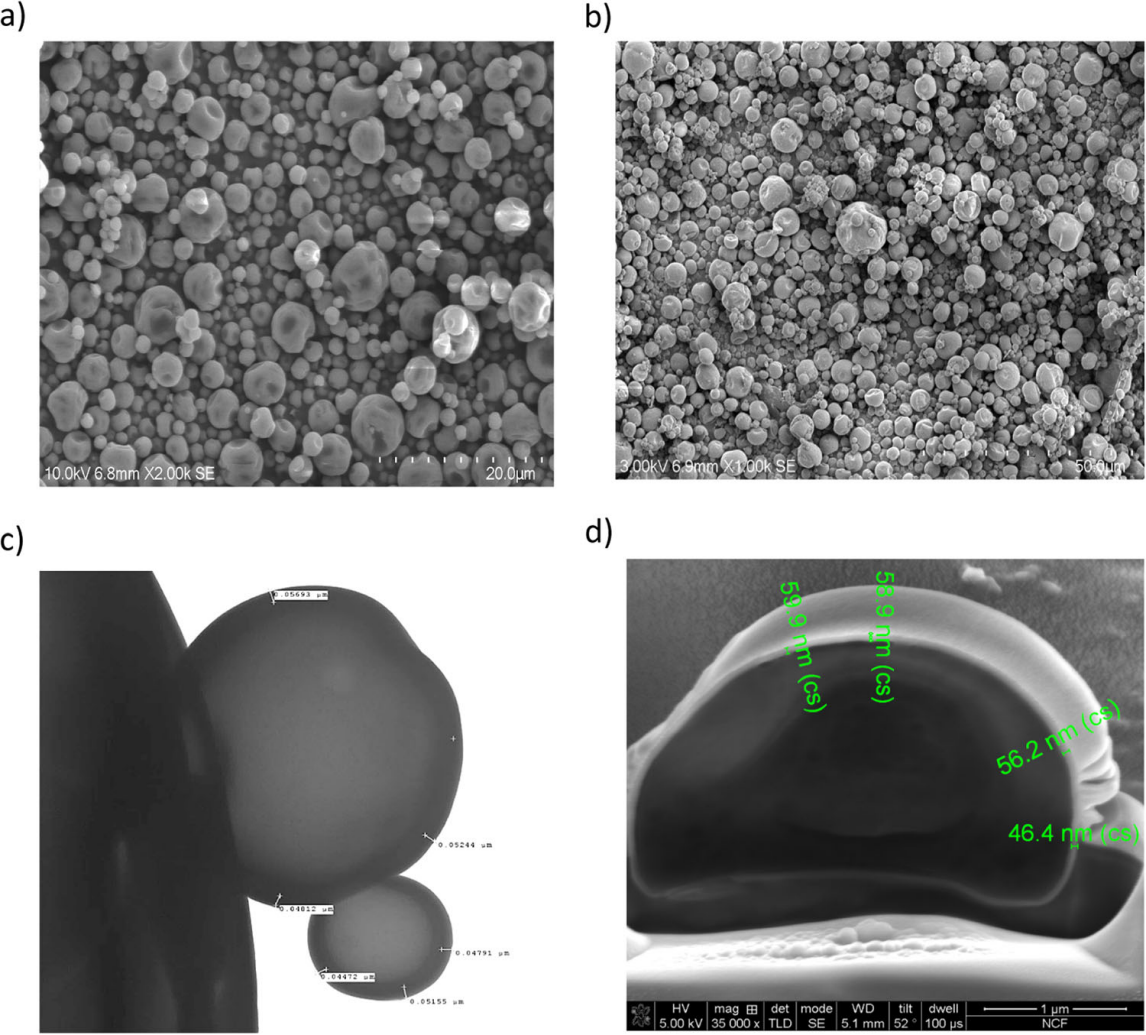
Particles visualized by various imaging technologies. **a** Spray-dried, uncoated particles imaged by SEM show a narrow particle size distribution and surface dimpling characteristics that were optimized by adjusting formulations to aid with fluidization during ALD. **b** Particles coated with 250 coats of alumina show no significant change in single particle morphology. **c** TEM image of particles with 250 ALD-alumina coats. Measurements confirm the value of 2.3 Å/ALD cycle as measured by ellipsometry. **d** Measurements using SEM after FIB milling of a particle coated with 250 coats of alumina confirm the value of 2.3 Å/ALD cycle.

**Fig. 2:**
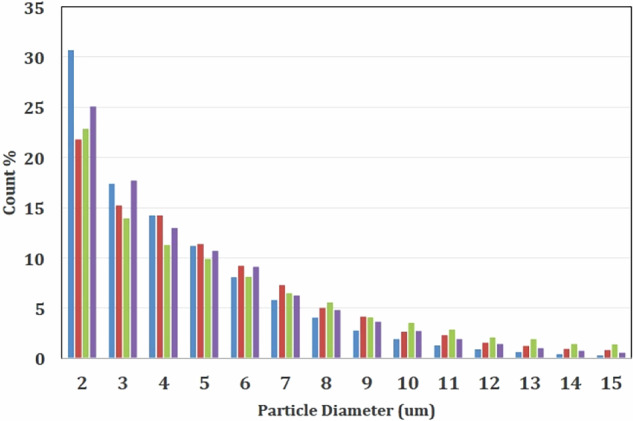
Particle size distribution of spray-dried and ALD-coated spray-dried particles as measured by flow imaging microscopy for particles with varying numbers of ALD-alumina coats. Left to right in each group of bars: Spray-dried, no coating (blue), 100 ALD cycles (red); 250 cycles, (green); 500 cycles (purple). Size distribution of the particles were estimated using the estimated spherical diameter (ESD) values returned by VisualSpreadsheet for each particle.

Glassy state vaccine powder formulations of capsomere antigens lyophilized with trehalose have been previously described^12^. In the current study, instead of using lyophilization to create glassy powders, mixtures of alum and HPV capsomere protein were spray-dried together with trehalose and hydroxyethyl starch added as glass transition temperature (*T*_g_) modifiers^20,21^. The resulting microparticles were spherical, with the majority of the particles ranging in diameter from 1 to 5 μm as measured by flow imaging microscopy (Figs 1a, 2). Hydroxyethyl starch added to the formulations raised the *T*_g_, and during drying also formed a “skin” that created dimple-like features on the particles as drying progressed. After additional drying, powders had a water content of <1% and corresponding *T*_g_ values above 100 °C that stabilized the protein against process temperatures and prevented particle agglomeration. The spherical geometry of the microparticles coupled with their surface dimpling promoted uniform fluidization during the subsequent ALD coating process (below) for adding defined numbers of alumina layers.

We then utilized ALD in a custom-built fluidized bed reaction chamber to apply conformal alumina coats of desired thickness to the spherical spray-dried microparticles. Alumina was deposited by injecting sequential pulses of trimethylaluminum vapor followed by water vapor in self-limiting reactions^15^ such that each cycle deposited one 2.3-Å-thick molecular layer of alumina on the microparticle surface. The fluidized bed reactor was operated at 70 °C, a temperature well below the *T*_g_ of the microparticles. Although the reactions for each half cycle were likely complete within 3–5 s, the duration of each coating cycle was set at ~2 min in order to allow purging of the system as monitored by a mass spectrometer between pulses. Even with this added time, the exposure time at 70 °C was far shorter than the month-long times over which HPV capsomere particles in these dried formulations are stable and maintain antigenicity^12^.

To determine the thickness and consistency of the ALD-alumina layers, two approaches were taken. First, silicon wafers were placed in the fluidized bed reactor, and coated co-currently along with spray-dried microparticles. These silicon wafers were then analyzed by ellipsometry, which showed that the thicknesses of the coatings were proportional to the number of coats applied (2.3 Å/coat, data not shown). The coated particles themselves were analyzed by scanning electron microscopy (SEM) for the overall particle morphology, by transmission electron microscopy (TEM) and focused ion beam milling–SEM for alumina layer thickness, gravimetry after calcining for alumina content, and by fluid imaging microscopy (FlowCam) for particle size distribution. Shown in Fig. 1 are examples of the spray-dried microparticles and their appearance after coating with 250 atomic layers of alumina, each layer with a thickness of approximately 2.3 Å, as visualized by SEM and focused ion beam-SEM. After spray-drying, particle sizes were distributed over a small range, and addition of up to 500 alumina coats did not appreciably affect this distribution, as expected given the relative sizes of the nanoscopic coatings and the micron-sized particles (Fig. 2). There were some fractured particles present, likely due to wall effects during fluidization in the current laboratory scale ALD reactor. Because these fractured particles have exposed antigen, we found that no additional antigen was required for a “prime” dose. With larger scale reactors and increased coating number, the percentage of fractured particles will likely decrease, thus necessitating addition of a priming amount of antigen to the particle preparation.

### In vivo release of injected coated antigens

**Fig. 3:**
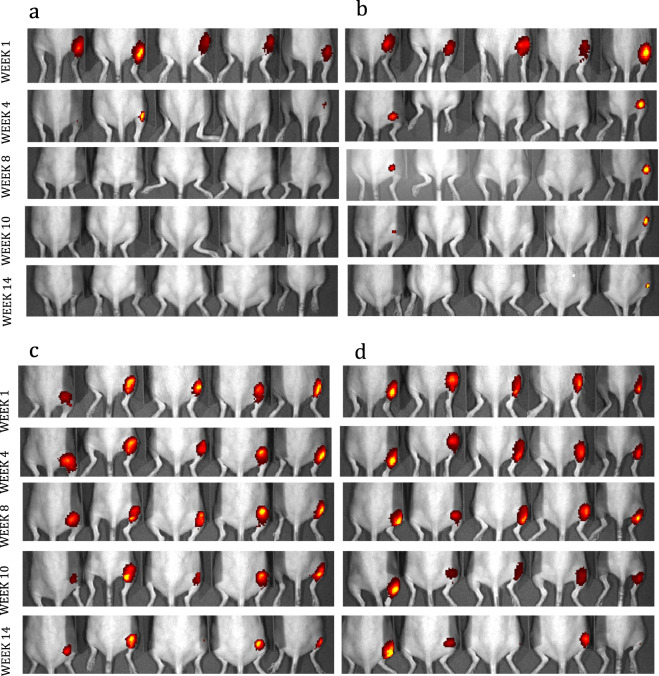
In vivo release of IR-dye labeled HPV16 L1 protein relative to the number of alumina layers applied to thermally stabilized microparticles. Fluorescent images of SKH1 mice recorded at weeks 1, 4, 10 and 14 following injection into their right dorsal thigh with **a** 5µg of HPV16 L1 that was labeled with IRDye 800CW and adsorbed to alum prior to spray-drying but not coated. **b** 5µg HPV16 L1 that was labeled with IRDye 800CW, adsorbed on alum, spray-dried and coated with 100 ALD-alumina layers. **c** 5µg HPV16 L1 that was labeled with IRDye 800CW, adsorbed to alum, spray-dried and coated with 250 ALD-alumina layers, and **d** 5µg HPV16 L1 that was labeled with IRDye 800CW, adsorbed to alum, spray-dried and coated with 500 ALD-alumina layers.

In order to characterize the in vivo release properties of the antigen from the alumina-coated microparticles, the HPV16 L1 protein was labeled with IR Dye 800CW prior to incorporation into ALD-coated microparticles. When injected into the hairless SKH1 mice, the labeled protein could then be tracked in vivo by a whole-body infrared detector. After injection into the hind limb, the mice were imaged weekly for up to 14 weeks, and the anatomic localization of uncoated labeled protein was temporally compared to that of protein coated with 100, 250, and 500 layers of alumina. Shown in Fig. 3 are scans at representative times after immunization visualizing the disappearance of the dye at the injection site for the different number of coats of ALD-alumina. The uncoated protein dispersed from the injection site over a period of 1–3 weeks, the protein with 100 coats at 4 weeks, and a fraction of the protein with 250 or 500 layers remained as a depot at the site of initial injection for almost 4 months. Although this imaging was not quantitative in that some fraction of the injected protein may leave the site undetected over time, it does indicate relative differences in antigen release from the particles in relation to number of alumina coats applied.

### Prime-boost immune kinetics of coated and liquid vaccine preparations

**Fig. 4:**
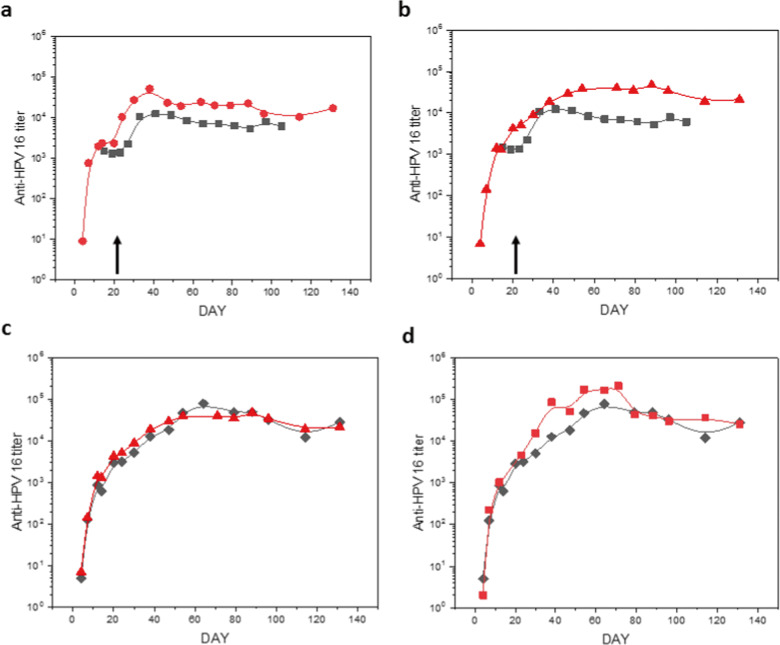
Antibody responses to vaccine formulations after immunization of BALB/c mice. Total anti-HPV16 antibody titers were measured by ELISA. Plots show geometric average (*n* = 10) of responses at each time point. **a** 5 µg prime/boost on days 0/21 with a suspension of HPV16 L1 capsomeres adsorbed on alum (indicated by black arrow; black squares) compared to similar spray-dried and reconstituted formulations (red circles). **b** 5 µg prime/boost on days 0/21 with a suspension of HPV16 L1 capsomeres adsorbed on alum (indicated by black arrow; black squares) compared to a single 10 µg immunization with alum-adsorbed spray-dried capsomeres with 250 coats of ALD-alumina on day 0 (red triangles). **c** Single 10 µg immunization with spray-dried capsomeres with 250 coats of ALD-alumina with (red triangles) and without (black diamonds) included alum. **d** Single 10 µg immunization with spray-dried capsomeres with 250 coats of ALD-alumina (black diamonds) and after incubation at 50 °C for 1 month (red squares).

**Fig. 5:**
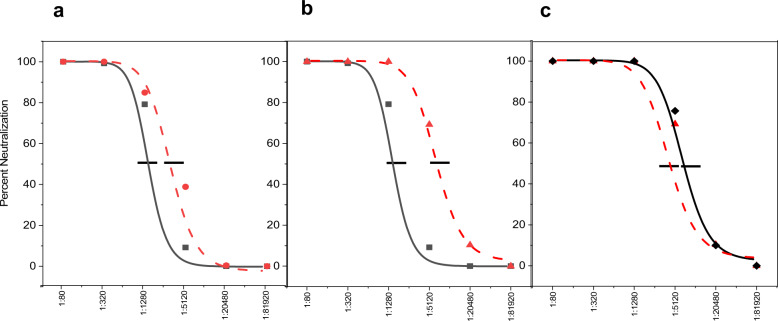
Comparison of neutralizing antibody responses for uncoated versus alumina-coated HPV16 L1 vaccine preparations. Neutralizing antibody responses (day 195 post prime) measured by percent neutralization of HPV16 pseudovirus (*n*=10) after **a** 5µg prime/boost immunization with liquid HPV16 L1 capsomeres plus alum (black squares, IC_50_ = 1600) or equivalent spray-dried formulations (red circles, IC_50_ = 4300), **b** 5µg prime/boost immunization with liquid HPV16 L1 capsomeres (black squares, IC_50_ = 1600) or a single 10µg dose of spray-dried HPV16 L1 capsomeres with 250 coats of alumina (red triangles, red triangles, IC_50_ = 13,800), and **c** single 10µg immunization with spray-dried HPV16 L1 capsomeres coated with 250 coats of alumina with (red triangles, IC_50_ = 12,300) and without (black diamonds, IC_50_ = 13,800) internal alum.

Two independent immunization studies of approximately 4 months duration each were performed to assess the variables of antigen concentration and the requirement for an internal alum adjuvant in eliciting a maximal immune response. The immunogenicities of HPV16 L1 vaccine formulations were determined both by ELISA to measure total anti-L1 antibodies and HPV16 pseudovirus neutralizing antibody assays of mouse sera at successive times after immunization. The antigen in the second experiment was concentrated fivefold in the spray-drying step while maintaining equivalent absolute amounts of antigen injected. As shown in Fig. 4a, b, both spray-dried and ALD-coated vaccine preparations were equivalent or superior in elicited antibody titers in comparison to formulations in solution with alum. These data confirmed that the thermostabilized particles were antigenically unaffected by the ALD process in a negative manner, and that overall the processes may have actually improved their immunogenicity. As shown in Fig. 4c, there was no difference in the antibody response observed whether or not alum was embedded within the glassy carbohydrate core of the particles. This result indicates that the alumina coating itself can serve as an adjuvant for the immune response, further reducing the required dosage of aluminum-based adjuvant. In Fig. 5 the neutralizing antibody responses basically paralleled the overall antibody measured by ELISA. These data confirm that combining the thermostabilization and ALD processes does not adversely affect conformation-specific epitopes required for the generation of neutralizing antibody responses. For the liquid prime-boost data, a clear plateau was observed in the titers at approximately 3 weeks post prime dose followed by an increase in titer after the boost. This plateau is also somewhat apparent in the coated preparations, although likely not as clear as might be observed if the time to boost was later.

### Thermostability of ALD-coated antigen microspheres

In previous work we showed that liquid formulations of Cervarix®, a commercial HPV vaccine, showed severe losses of antibody and neutralizing antibody titers after 3 months incubation at 50 °C, whereas titers for lyophilized, adjuvanted capsomere vaccines were unchanged following incubation^12^. In the current study, HPV16 L1 vaccines formulated as spray-dried microparticles and as ALD-coated microparticles were incubated for 1 month at 50 °C before testing in mice. As shown in Fig. 4d, storage at this temperature did not affect antibody response to the vaccine.

## Discussion

We have developed a vaccine preparation technology that potentially will enable “single-shot” immunization for a variety of vaccine antigens. This platform uses a highly scalable molecular deposition process to create thermostable microparticles with coatings that not only serve as adjuvants, but also deliver temporally separated primer and booster vaccine doses from a single injection. Previously we reported how trehalose-antigen formulations could be thermostabilized via lyophilization. In this current study the antigen-trehalose solutions instead were spray-dried to yield spherical microparticles. The thermostable properties of these microparticles then enabled their coating at temperatures approximating 70 °C, in short cycle times. This spherical geometry also allowed ALD to be conducted in fluidized bed reactors, depositing coatings uniformly on the surface of the particles. The ability to modulate the number of coating layers enables a tunable boost time.

The current reactor is at laboratory scale, and we noted a small fraction of broken or incompletely coated microparticles at early cycle numbers. These broken particles likely result from the small chamber size, and interactions with the chamber walls during fluidization. We expect that as the reaction chamber increases in volume, these effects will be minimized. In any case, at higher layer numbers, the fraction of incompletely coated particles decreases. For the current experiments we have actually utilized the presence of the broken particles (which release their dose instantly upon injection) as the priming dose. We anticipate that as coating improves, a separate priming dose may have to be added to the formulation.

We measured both total and neutralizing antibody responses generated against the HPV capsomere preparations. Such antibodies have been previously shown to be the dominant protective immunologic responses to HPV capsid antigens^22,23^. The major differences in antibody responses between the experimental groups were (1) an ≈3–8-fold increase in titers for the ALD-coated versus uncoated antigen preparations, (2) no apparent adjuvant advantage for including additional alum within the spray-dried core of the particles, i.e., the ALD coating itself provided adequate adjuvanting effect, (3) a more sustained depot effect at the site of injection for ALD-coated particles compared to that for conventional liquid suspensions of antigen adsorbed on alum, and most importantly (4) a boost response from a single administration of the coated preparation. These differences were observed in two independent immunization studies that achieved almost identical titers and temporal responses between the groups.

The ALD process is very flexible with respect to potential vaccine applications. Because the time-release characteristics depend on the number of layers applied, the time between prime and boost doses can be controlled by applying precise numbers of coating layers. Fluidized bed reactors are well-established in the chemical process industry, and offer facile scaling to produce bulk quantities of coated powders. We have examined alumina coatings because of the substantial historical use of aluminum oxide adjuvants, but a vast number of ALD-compatible chemistries are available for deposition of other metal oxide (e.g., titania, silica) and organometallic (e.g., aluminum alkoxide) layers. Many potential adjuvants (e.g., monophosphoryl lipid-A) could be included within the glassy particle cores, and coated microparticles containing a variety of included antigens might be combined in a single formulation allowing their simultaneous administration. Other advantages of ALD coatings aside from the time-release kinetics of the immune response include protection from humidity^24^ and the ability to mix combinations of different antigens that might otherwise be incompatible in liquid formulations.

Simplifying the immunization schedule to a single dose/administration will correspond to cost reductions through logistics, personnel, and supply variables associated with repeated immunizations, as well as cold-chain/storage costs through improved vaccine thermostability. Moreover, we anticipate that this platform would require less total antigen than current multiple single dosing modalities, could be readily applied to a number of antigens (or multiple antigens), have flexibility for the incorporation of improved adjuvants, and could be readily scaled and adapted to vaccine production “in country” thus ensuring sustainability.

## Methods

### Reagents

All chemicals were reagent grade. High purity, low endotoxin α,α-trehalose dihydrate was a generous donation of Pfanstiehl (Waukegan, Illinois). Two percent Alhydrogel® (aluminum hydroxide adjuvant) was obtained from Accurate Chemicals and Scientific Corp (Westbury, NY). 3,3′,5,5′-tetramethylbenzidine (Turbo TMB) and peroxidase-conjugated donkey anti-mouse IgG (H + L) was from Thermo Scientific (Rockford, IL). Plasmid-safe DNase was from Epicentre (Madison, WI). Hydroxyethyl starch/Viastarch (HES) was obtained from Fresenius Kabi, Austria, GmbH. IR Dye 800CW NHS was obtained from LI-COR Biosciences, Bad Homburg, Germany.

### HPV16 L1 capsomere protein purification

The HPV16 L1 protein was purified as a non-GST fusion protein with deletions at both its amino and carboxy termini (capsomeres)^12^. Briefly, HPV16 L1 was expressed in HMS174 competent *E. coli* (Millipore Sigma, St. Louis, MO, P/N 69452-M) culture. The bacteria were pelleted and lysed at 800–1000 bar in a GEA Niro Soavi Panda homogenizer (Bedford, NH). The soluble fraction was collected and the L1 precipitated using 30% ammonium sulfate. Following re-homogenization of the precipitate at 500 bar (Panda), the protein was chromatographed on a Q High Performance sepharose anion exchange column (GE Healthcare, Piscataway, NJ). L1 was eluted as pentamers from the sepharose column using a sodium chloride gradient. A final purity of >95% was estimated by SDS-PAGE. Capsomere preparations were tested for endotoxin using a QCL 1000TM Limulus Amebocyte Lysate test kit (LONZA, Basel, Switzerland), and found to contain <1 EU/mL. Before formulation, fractions containing L1 were exchanged by size exclusion chromatography into a 100 mM histidine buffer pH 7.1.

### Fluorescent dye labeling of HPV16 L1 capsomeres

Labeling of HPV16 L1 capsomeres with IRDye® 800CW NHS ester was performed according to the manufacturer’s instructions, using a protein concentration of 1 mg/mL in 1× phosphate-buffered saline (PBS) pH 8.5 and dye added according to the molecular weight of the L1 protein, so that the molecular ratio of dye to protein was between 1:3 and 1:3. The dye and protein mixture was allowed to react for 2 h at 20 °C, protected from light, and gently mixed by end-over-end rotation. Labeled capsomeres were transferred to a Zeba desalting spin column to remove excess dye and exchanged into 100 mM histidine pH 7.1 for formulation. The final labeled HPV16 L1 capsomere concentration was ≈0.7 mg/mL. The dye-to-labeled-protein ratio was calculated to be 1:2 using the absorbance of the final product at the excitation maxima of the dyes and protein.

### Preparation of spray-dried vaccine formulations

Prior to spray-drying, 0.5 mg/mL HPV16 L1 capsomeres (labeled either with IRDye 800CW for the biodistribution study, or unlabeled for the immunogenicity study) were formulated in 54 mM histidine HCl with 15 w/v% endotoxin-free trehalose, 2.5% w/v HES, 40 mM NaCl, 0.02 mM Tween 80. Some formulations also contained 0.5 mg/mL aluminum from Alhydrogel® (alum) with a final pH of 6.0. Alum-containing formulations were rotated end over end in 50 mL polypropylene centrifuge tubes at 4 °C for 1 h to allow adsorption of capsomeres to the alum adjuvant. All formulations were spray-dried in a Buchi B-290 Mini Spray Dryer (Buchi Labortechnik AG, Flawil, Switzerland) fitted with a two-fluid nozzle. Particles were collected in a high-performance cyclone separator and yields were calculated to be ≥80% based on formulation solid content. Water content was measured by Karl-Fischer titration to be approximately 5%. Particles were further dried in a lyophilizer (FTS Systems Lyophilizer, Warminster, PA) at 60 Torr for 16 h at 40 °C. Pressure was brought up to 640 mTorr and the vials were backfilled with nitrogen and sealed. Water content following this further drying was determined by Karl-Fischer analysis. *T*_g_ values for the particles were determined by differential scanning calorimetry.

### Particle size analysis

Particle size was measured using a FlowCam VS system (Fluid Imaging Technologies, Inc., Scarborough, ME). The instrument used a 100-μm flow cell and a 10× objective to image particles between 1 and 30 µm. The flash duration was set so that the average pixel intensity of the background was between 150 and 160. Before use, the flow cell was cleaned with 1% Hellmanex III solution and ultrapure water. The instrument was focused using the default autofocus procedure on 20-μm calibration beads. One milligram of each particle sample was resuspended with 250 μL of ethanol (200 proof) for each measurement and each sample was measured at least three times. The flow cell was flushed with ultrapure water between measurements. AutoImage mode was used to collect images at a rate of 20 s^−1^ and 30% efficiency. Size distribution(s) of the particles were estimated using the estimated spherical diameter (ESD) values returned by the VisualSpreadsheet software for each particle. These diameters were then grouped into bins to construct the size distribution.

### Atomic layer deposition (ALD)

Particles were coated with alumina (aluminum oxide, (Al_2_O_3_)) layers by ALD in a custom-built, low pressure fluidized bed reactor. The alumina layers were formed by alternating exposure to TMA (trimethylaluminum, Al(CH_3_)_3_) and water vapors under argon at 70 °C and 2–3 Torr. An online mass spectrometer was used to monitor concentration of the methane byproduct of the ALD reactions, as well as concentrations of any unconsumed TMA and water. The TMA/H_2_O cycles were repeated between 100 and 500 times to obtain the desired thickness. Even and uniform coatings on all sides of the particles were maximized by fluidization with a constant argon stream and agitating the reactor with a custom-built eccentric weight vibrator. To further decrease agglomeration, the reaction was interrupted every 70−100 cycles and the particles were sieved to remove or break agglomerates.

The thickness of the alumina layers was estimated by FIB milling, TEM imaging and by the alumina mass fraction of the coated particles that could be determined by calcining at 600 °C. Growth rates of 2.3 Å/cycle were within the range of the literature. After coating with 250 coats of alumina, powders contained 3 mg capomeres per gram powder.

### ALD film analysis

A spectroscopic ellipsometer (J. A. Woollam Co., Lincoln, NE) was used to determine the thickness and refractive index of Al_2_O_3_ films deposited onto silicon wafers inserted into the fluidized bed reactor chamber. The thickness was measured at 550 nm at three different angles of incidence: 60°, 70° and 80°. Refractive indexes of deposited Al_2_O_3_ were obtained using the Al_2_O_3_ and Cauchy models. The final thickness was obtained by averaging the Al_2_O_3_ thickness values obtained over multiple reactor runs.

### Scanning electron microscopy

Coated and uncoated particles were mounted on imaging stubs using double-sided adhesive carbon tape, sputtered with platinum for 15 s and imaged with an accelerating voltage of 5 kV on a Hitachi SU3500 Variable Pressure SEM (Hitachi High-Technologies America, Inc).

### Focused ion beam (FIB) milling

Coated particles were mounted and sputtered as in SEM. Once particles were loaded into the FEI Nova NanoLab 600 DualBeam (FIB/SEM) System, an additional platinum mask was deposited locally by a focused Ga ion beam at 30 kV and 28 pA to an approximate thickness of 0.1 µm. An ion beam at 30 kV and 93 pA was used to create a cross-sectional wall into a selected particle. SEM images were taken at 5 kV and 98 pA with the TLD (through-lens detector) in the SE (secondary electron) mode.

### Transmission electron microscopy

Coated particles were placed on formvar/carbon-coated, glow-discharged 400 mesh copper TEM grids. Images were collected using an FEI Tecnai T12 Spirit TEM operating at 100 kV and an AMT 2k × 2k CCD. Measurements were made with the AMT camera software.

### Vaccine immunogenicity

Murine immunogenicity studies were conducted under the University of Colorado at Boulder Institutional Animal Care and Use Committee (IACUC) protocol #2318. Five HPV16 L1 capsomere formulations (see legend, Fig. 4 for injection schedule) were tested in female BALB/c mice from Taconic (Hudson, NY). Mice were allowed to acclimate at least 1 week before use and were 10–11 weeks old at the start of the study. Eight mice were used in each group. Mice were injected intramuscularly (i.m.) into the right dorsal thigh on days 1 and, for some groups, also on day 22 (see legend, Fig. 4). Uncoated, spray-dried samples were reconstituted in water for injection prior to administration. ALD-coated particles were suspended in 54 mM histidine HCl with 15 w/v% endotoxin-free trehalose, 2.5% w/v HES, 40 mM NaCl, 0.02 mM Tween 80 immediately prior to injection.

Blood samples were collected from the sub-mandibular artery under isoflurane anesthesia every 8 days. Serum was separated by centrifugation at 4000 × *g* for 6 min at room temperature and stored at −80 °C until use.

### Antigen distribution and release

Live animal antigen imaging studies were conducted at the University of Colorado Anschutz Animal Imaging Core, a facility of the University of Colorado Cancer Center. Female SKH1 mice from Charles River Laboratories (Lentilly, France) were aged 10–11 weeks old at the start of the immunization study. IRDye® 800CW-labeled HPV16 L1 capsomeres were formulated at 0.1 mg/mL with 0.5 mg/mL Al (as Alhydrogel) in a buffer solution containing 9.38 wt.% trehalose, 1.56 wt.% HES, 33.8 mM histidine, 25 mM NaCl and 0.02 mM Tween80. The formulation was spray-dried to form microparticles, and then divided into four fractions. 100, 250, or 500 ALD-alumina coatings were applied to three fractions; the uncoated remainder was retained as a control. Immediately prior to injection into mice, uncoated particles were reconstituted with water for injection and ALD-coated particles were suspended in an isotonic buffer solution containing 9.38 wt.% trehalose, 1.56 wt.% HES, 33.8 mM histidine, 25 mM NaCl and 0.02 mM Tween80. In all cases, the final concentration of IRDye® 800CW-labeled HPV16 L1 capsomeres was 0.1 mg/mL. Fifty microliters of the formulations was administered intramuscularly into the right dorsal thigh of the mice. Each formulation was injected into a group of five mice. In vivo fluorescence imaging was captured with the IVIS Xenogen200 imaging system (PerkinElmer). The mice were anesthetized with 2% of isoflurane and group images were taken with mice in the ventral position. The images were captured using Living Image software. Mice were imaged immediately, 1 day, 3 days and 1 week after injection, and then weekly for an additional 5 weeks. Blood samples were collected from the sub-mandibular artery while under isoflurane anesthesia for imaging on days starting with day 14 and ending on day 70. Serum was separated by centrifugation at 4000 × g for 6 min at room temperature and stored at −80 °C until use.

### Detection of L1 antibodies

An ELISA was used to detect anti-HPV16 L1 titers in mouse sera. HPV16 L1 was adsorbed onto Nunc 96-well flat bottom PolySorp Immuno plates and incubated overnight at 4 °C. The next day, plates were blocked (5% non-fat dry milk, 0.05% Tween 20 in PBS) for 1 h at 37 °C. Mouse sera was then added and diluted across the plate. Plates were incubated for 1 h at 37 °C. Following incubation, an anti-mouse HRP-conjugated IgG antibody was added and plates were incubated at 37 °C for 1 h. Ultra TMB was added and plates were incubated at room temperature for 1 min after which the reaction was quenched with 1 M H_2_SO_4_. Absorbance was measured at 450 nm on a BioTek Microplate Reader (Winoosky, VT). To determine titers, average OD 450 values as a function of dilution were fit to a four-parameter logistic equation using a Python script. Cutoff values were determined by assaying naïve bleeds.

### Pseudovirus production and neutralizing antibody determination

To prepare pseudovirions, 293TT cells (obtained from Chris Buck, National Cancer Institute) were transfected with DNA plasmids expressing secreted alkaline phosphatase (SEAP), HPV16 L1 and HPV16 L2 capsid proteins^12^. Cells were chemically lysed 2–3 days after transfection. The pseudovirions were salt extracted and isolated by sedimentation in an Optiprep™ gradient. Fractions eluted from the Optiprep™ gradient were assayed for DNA and protein content with PicoGreen and BCA assays, respectively.

To determine neutralizing antibody titers, 293TT cells were plated in 96-well plates and incubated at 37 °C for 2–5 h. Mouse sera was diluted in separate 96-well U-bottom plates. HPV16 pseudovirus was then added to the sera dilutions and allowed to incubate on ice for 1 h. The pseudovirus-mouse serum solution was added to the plated 293TT cells and incubated at 37 °C for 3 days. Following incubation, the supernatant from the cells was collected and assayed for the presence of SEAP using The Great Escape SEAP Chemiluminescence test kit (Clontech, Mountain View, CA). Plates were read using a BioTek luminometer (Winoosky, VT). Neutralizing antibody titers were defined as the dilution of mouse serum that neutralized 50% of the pseudovirus signal as determined by the SEAP fluorometric measurement^12^.

### Reporting summary

Further information on experimental design is available in the Nature Research Reporting Summary linked to this paper.

## Supplementary information

Reporting Summary

## Data Availability

All datasets used and/or analyzed in the current study are available from the corresponding author upon reasonable request.
