# Author Correction: Single-administration, thermostable human papillomavirus vaccines prepared with atomic layer deposition technology

**DOI:** 10.1038/s41541-025-01363-y

**Published:** 2026-01-12

**Authors:** Robert L. Garcea, Natalie M. Meinerz, Miao Dong, Hans Funke, Saba Ghazvini, Theodore W. Randolph

**Affiliations:** 1 The BioFrontiers Program, University of Colorado, Boulder, CO USA; 2 Department of Molecular, Cellular, Developmental Biology, University of Colorado, Boulder, CO USA; 3 Department of Chemical and Biological Engineering, University of Colorado, Boulder, CO USA

**Keywords:** Biotechnology, Immunology

Correction to: *npj Vaccines* 10.1038/s41541-020-0195-4, published online 02 June 2020

In the original Article, Figure 2 was incorrect and did not include the spray-dried particle size distribution. The correct Figure 2 is provided below.

Incorrect Figure 2
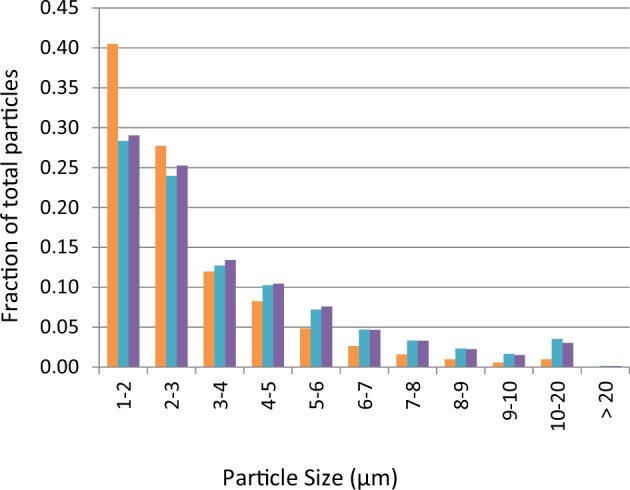


Correct Figure 2
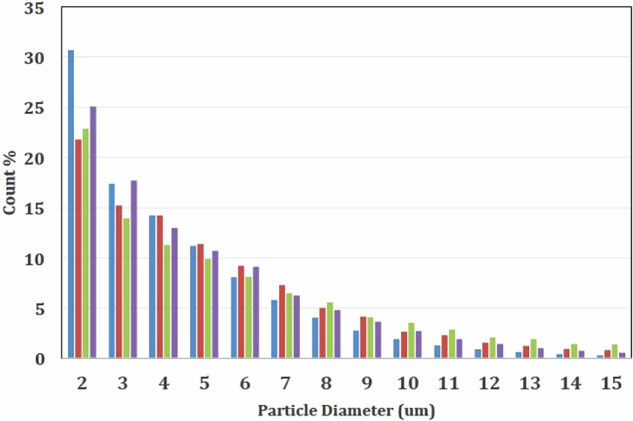


The original article has been corrected.

